# Efficacy of Yougui pill combined with Buzhong Yiqi decoction in alleviating the sexual dysfunction in female rats through modulation of the gut microbiota

**DOI:** 10.1080/13880209.2021.2010774

**Published:** 2021-12-14

**Authors:** Yangyun Wang, Chaoliang Shi, Wandong Yu, Wei Jiao, Guowei Shi

**Affiliations:** Department of Urology, The Fifth People’s Hospital of Shanghai, Fudan University, Shanghai, China

**Keywords:** Sexual function, ovariectomized rat, traditional Chinese medicine, ELISA, 16S rRNA sequencing

## Abstract

**Context:**

Yougui pill combined with Buzhong Yiqi decoction (YPBYD) is used to relieve sexual dysfunction in clinical practice.

**Objective:**

To investigate changes in microbial composition caused by sexual dysfunction and identify dominant bacteria related to YPBYD treatment.

**Materials and methods:**

Female Sprague-Dawley rats were randomly divided into four groups (*n* = 6): one group underwent Sham operation (Sham group), while three groups underwent ovariectomy (one model and two treatment groups). The ovariectomized (OVX) rats received oestradiol benzoate (250 µg/kg/week) or YPBYD (3.6 mL/d) via oral gavage for 4 weeks. Vaginal smear assay was performed; the serum levels of cyclic adenosine monophosphate (cAMP) and oestradiol (E2) were measured, followed by collection of stool samples for 16S rRNA sequencing.

**Results:**

After YPBYD treatment, the levels of E2 and cAMP in OVX rats significantly increased (E2: from 20.45 ± 1.60 ng/L to 24.38 ± 1.70 ng/L; cAMP: from 261.41 ± 9.21 pg/mL to 373.75 ± 17.37 pg/mL). OVX treatment decreased diversity of gut microbiota and YPBYD treatment restored gut microbiota composition. Compared with Sham group, the abundance of *Romboutsia* significantly increased, while those of *Proteobacteria* and *Staphylococcus* markedly decreased in OVX group (all *p* < 0.05); meanwhile, the abundance of these microbes showed an opposite trend after YPBYD treatment. These microbiotas were involved in tyrosine and tryptophan biosynthesis and fatty acid metabolism.

**Discussion and conclusions:**

These findings are the first to indicate YPBYD can alleviate female sexual dysfunction by modulating gut microbiota in OVX rats, which will help enhance the understanding on potential mechanism of YPBYD against sexual dysfunction.

## Introduction

A human sexual response is the result of the complex interaction between biological and social psychological factors; sexual interaction is the physical manifestation of our need for acceptance and our need for life (Clayton and Valladares Juarez 2017). Therefore, sexual function plays an important role in improving an individual’s quality of life (QoL) and subjective well-being. However, the prevalence of sexual dysfunction in men and women increases with age (Stringer 2016). Dysfunction usually involves desire disorders, arousal disorder, dyspareunia and orgasmic disorder (McCabe et al. 2016). It has serious adverse effects on the QoL of patients and their sexual partners, such as marital discord, diminished self-esteem and psychological distress (Chung et al. 2015); hence, it is necessary to carry out an effective clinical treatment for sexual dysfunction.

Several synthetic medications (sildenafil and bremelanotide) (Berry and Berry 2013) are used in the management of male or female sexual dysfunction. However, these treatments are associated with some side effects (such as nausea, flushing and urinary tract infection) and economic problems (expensive cost) (Ohl et al. 2017; Simon et al. 2019). Based on these factors, traditional Chinese medicine (TCM) has attracted the attention of researchers as it is a natural form of medicine with fewer side effects, and can be made up of easily available properties (Chubak and Doctor 2018).

TCM has been used widely to treat sexual dysfunction in China for more than 2000 years, which significantly improves the sexual function of patients with sexual dysfunction (Chubak and Doctor 2018). TCM believes that the onset of sexual dysfunction is related to the imbalance of Yin-Yang in the kidney and liver, and is combined with Qi-deficiency and blood-stasis (Wei and Wei 2019). Thus, from the perspective of TCM, tonifying kidney, invigorating spleen and promoting blood circulation are the basic principles of sexual dysfunction treatment (Guo et al. 1999; Geng et al. 2019). Yougui pill and Buzhong Yiqi decoction are two important TCMs.

Among these, Yougui pill has been widely used to recuperate kidney-yang deficiency syndrome clinically for 400 years in China (Chen et al. 2019). Buzhong Yiqi decoction has the effects of raising Yang, removing blood stasis and strengthening the spleen and stomach (Lu et al. 2021). Taken together, Yougui pill combined with Buzhong Yiqi decoction (YPBYD), which consists of 17 traditional Chinese medical herbs, is effective in relieving sexual dysfunction in clinical practice (Liu et al. 2015; Wang 2015). Moreover, our previous study revealed the molecular mechanism of YPBYD in the treatment of sexual dysfunction from the perspective of network pharmacology (Wang et al. 2019). Research has indicated that the microbiota has many important functions, which can affect human development, physiology and emotion. The microbiota also communicates with the central nervous system, affecting brain function and behaviour (Mayer et al. 2015). Based on the relationship between microbiota and human diseases, Tirandaz et al. (2018) suggested that microbiota manipulation might be a potential treatment for sexual dysfunction via improving sexual behaviour. However, the direct evidence that YPBYD regulates the intestinal microbiota to improve the symptoms of sexual dysfunction is limited.

Hence, this study investigates the changes in the microbial composition caused by sexual dysfunction and identifies the dominant bacteria targeted by the YPBYD treatment. First, we established the sexual dysfunction model of female rat. Next, the oestrous cycle of rats was observed to evaluate whether the model was successfully constructed. Finally, the stool samples of rats were collected for 16S rRNA sequencing. This study will provide a theoretical basis for evaluating the efficacy of YPBYD in treating female sexual dysfunction through the regulation of the gut microbiota structure, and help researchers to further understand the interaction between the host and microorganisms during the treatment of sexual dysfunction.

## Materials and methods

### Animals

Eight-week-old female Sprague-Dawley rats (200 ± 20 g) were purchased from Shanghai Jihui Experimental Animal Feeding Co., Ltd. (Shanghai, China). All rats were maintained under standard laboratory conditions (18–22 °C) with 50–60% humidity for 1 week prior to the experiment, with *ad libitum* access to water and chow diet. This study was approved by the Animal Ethics Committee of Shanghai Fifth People’s Hospital, Fudan University, and all protocols were performed in accordance with the National Institutes of Health Guidelines for the Care and use of Laboratory Animals.

### Preparation of YPBYD

YPBYD was composed of 17 different herbs, including *Rehmannia glutinosa* (the steamed and sun-dried tuber of *Rehmannia glutinosa* (Gaertn.) Libosch. ex Fisch. et Mey. [Scrophulariaceae], 24 g), Chinese yam (the dried root of *Dioscorea opposita* Thunb. [Dioscoreaceae], 30 g), Corni Fructus (the fruit of *Cornus officinalis* Sieb. et Zucc. [Cornaceae], 15 g), barbary wolfberry fruit (the fruit of *Lycium chinense* Miller (Solanaceae), 9 g), Dodder Seed (the seed of *Cuscuta chinensis* Lam. [Convolvulaceae], 12 g), deerhorn glue (the gelatine made from the horns of *Cervus nippon* Temminck [Cervidae], 12 g), Eucommia bark (the stem bark of *Eucommia ulmoides* Oliver [Eucommiaceae], 12 g), cinnamon (the bark of *Cinnamomum cassia* Presl (Lauraceae), 6 g), Chinese angelica (the root of *Angelica sinensis* (Oliv.) Diels [Umbelliferae], 9 g), aconite (the root of *Aconitum carmichaeli* Debx. [Ranunculaceae], 10 g), Radix Astragali (the dried root of *Astragalus membranaceus* (Fisch.) Bge. [Leguminosae], 18 g), liquorice (the root and rhizome of *Glycyrrhiza uralensis* Fisch. [Leguminosae], 9 g), ginseng (the fleshy root of *Panax ginseng* C. A. Meyer [Araliaceae], 6 g), orange peel (the peel of *Citrus reticulata* Blanco [Rutaceae], 6 g), Morinda root (the root of *Morinda officinalis* How. [Rubiaceae], 15 g), Atractylodes (the rhizomes of *Atractylodes lancea* (Thunb.) DC. [Compositae], 9 g) and Herba Epimedii (the aerial part of *Epimedium brevicornum* Maxim. [Berberidaceae], 15 g). The YPBYD solution was prepared as follows: dried Chinese medicinal materials were soaked in 800 mL of water for 30 min and then boiled for 30 min to obtain the concentrated YPBYD potion.

### OVX model construction and treatment

All rats were randomly divided into four groups (six rats per group): Sham group, ovariectomized (OVX) group, OVX treated with oestradiol benzoate group (OVX + EB) and OVX treated with YPBYD group (OVX + YPBYD). The approach of model construction was described previously (Xu et al. 2019). In brief, the rats in each group were anaesthetized by intraperitoneal injection of 1% pentobarbital sodium (40 mg/kg). In the Sham group, a longitudinal incision was made on the abdominal wall of the rats, and the fat around the ovary was removed. In OVX rats, bilateral ovaries were excised through the longitudinal incision. Within four days after surgery, the rats in the OVX + EB group were subcutaneously injected with 250 µg/kg of EB once a week. Seven days after surgery, the rats in the OVX + YPBYD group were administered with 3.6 mL of YPBYD via oral gavage once a day for four consecutive weeks. The dose was calculated based on the ratio of human and animal body surface area with a coefficient of 0.018. The rats in the Sham and OVX groups were injected with the same amount of vegetable oil. After treatment for 4 weeks, all animals were euthanized. The blood samples were collected before the rats died. Meanwhile, the stool samples were collected for subsequent experiments.

### Vaginal smear assay

Vaginal smears were taken on the 5th day after surgery (once a day for five consecutive days) to observe the characteristics of vaginal cell changes during the oestrus cycle in each group. The vaginal contents were collected using a cotton swab moistened with normal saline by inserting the tip into the rat vagina within 0.5 cm, and then the vaginal fluid was evenly spread on the glass slide. After natural drying, the slides were fixed in 10% formalin solution for 10 min and stained with Giemsa solution for 10 min. Next, the morphology of vaginal cells was observed under a microscope. Notably, three types of cells were detected on the vaginal smears: keratinocytes (polygonal cells without nucleus); leukocytes (small and round cells); and epithelial cells (round nucleated cells). The stage of the oestrus cycle was determined based on the ratio of these cells. Specifically, the oestrus cycle of rats is four days and is divided into five stages: pro-oestrus, oestrus, metoestrus I, metoestrus II and dioestrus.

### Enzyme-linked immunosorbent assay

Blood was collected from the abdominal aorta of rats and then centrifugation at 1600×*g* for 15 min at 4 °C to obtain serum samples. The concentration of oestradiol (E2) and cyclic adenosine monophosphate (cAMP) was measured by enzyme-linked immunosorbent assay (ELISA) kits (E2: CGE20048, Shanghai Chen Gong Biotechnology Co., Ltd., Shanghai, China; cAMP: B20344, Jianglaibio Co., Ltd., Shanghai, China) according to the manufacturer’s protocol.

### Bacterial DNA extraction and 16S rRNA sequencing

The total bacterial DNA was extracted from the stool samples of each rat using the cetyl trimethyl ammonium bromide/sodium dodecyl sulphate (CTAB/SDS) method. The purity and concentration of DNA were detected utilizing a NanoDrop 2000 spectrophotometer, while the DNA integrity was detected by 1% agarose gels. DNA with A260/A280 ratio between 1.8 and 2.0 was considered qualified. 16S rRNA sequencing was performed as previously described (Wu et al. 2016). In brief, the V3–V4 region of the 16S rRNA gene was amplified by polymerase chain reaction with 338F (ACTCCTACGGGAGGCAGCAG) and 806R (GGACTACHVGGGTWTCTAAT) primers. Then, the libraries were generated and sequenced on an Illumina HiSeq2500 platform. Finally, 250 bp paired-end reads were obtained.

### Analysis of sequenced data

After sequencing, the paired raw data were merged using Trimmomatic (Bolger et al. 2014). Quality control of raw data was performed; then, the data were filtered into clean reads using the Quantitative Insights Into Microbial Ecology software (http://qiime.org/). Based on the similarity level, the sequences were divided into different operational taxonomic units (OTUs); OTUs with ≥97% similarity level were selected for bioinformatics analysis using USEARCH (version 7.1, http://drive5.com/uparse/). Taxonomic analysis of OTUs with a similarity level of greater than 97% was performed using RDP classifier (http://sourceforge.net/projects/rdp-classifier/) to obtain the community composition of each sample. The rank abundance curve and rarefaction curve were plotted to determine the species abundance and species uniformity.

### Diversity analysis

Next, alpha-diversity indexes including ACE, Chao, Shannon and Simpson were calculated to access the richness and diversity of species. The beta-diversity was also analysed to display the similarity of gut bacteria composition between different samples. Results were visualized using principal component analysis (PCA), principal coordinate analysis (PCoA) and nonmetric multidimensional scaling (NMDS).

### Differential abundance analysis and function prediction

To identify the altered microbiotas related to OVX and drug treatment, the differential abundance analysis of microbes was performed. The Kruskal–Wallis (KW) rank sum test is a method used to perform a non-parametric test of multi-group independent samples, which can analyse the significant difference of species in multiple groups and calculate the false discovery rate *q* value for the *p* value (Guo et al. 2013). In this analysis, we used KW rank sum test to evaluate the differential abundance among the four groups. Moreover, linear discriminant analysis effect size (LEfSe) (Segata et al. 2011) was applied to determine the microbiotas that could best represent the characteristics of each group; then, a linear discriminant analysis (LDA) was used to estimate the impact of the abundance of each microbe on the different effect. The taxa with a higher LDA score indicated a higher consistency; the taxa with an LDA score of >2 and a *p* value of <0.05 were considered significant. Furthermore, phylogenetic investigation of communities by reconstruction of unobserved states (PICRUst) (Douglas et al. 2018) was used to predict the functional enrichment of different microbes.

### Statistical analysis

All data were expressed as mean ± standard deviation and were analysed using one-way analysis of variance. Statistical analysis was performed using GraphPad prism 5 (GraphPad Software, San Diego, CA). A *p* value of less than 0.05 was considered significant.

## Results

### Effect of YPBYD on the oestrus cycle

**Figure 1.**
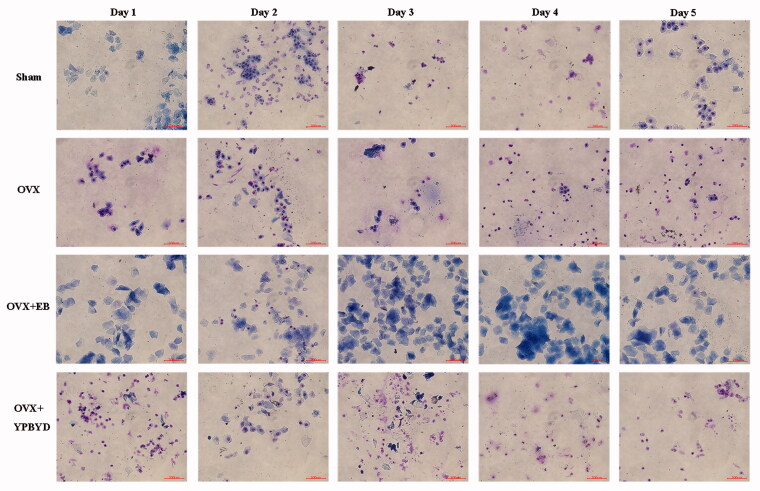
Effect of YPBYD treatment on the oestrous cycle of rats in each group. Representative images of vaginal epithelium cells taken under ×200 magnification. The nucleus is stained purple or blue purple, and the cytoplasm is stained pink.

To verify whether the model was successfully constructed, we observed the oestrous cycle of rats in each group. Most of the cell types in the oestrous dioestrus state were leukocytes, those in the pro-oestrus state were epithelial cells, those in the oestrus state were keratinocytes, and those in the late oestrus state were keratinized cells and leukocytes. As shown in Figure 1, the rats in the Sham group showed a complete oestrus cycle. The vaginal epithelial cells of rats in the OVX group were always composed of leukocytes, indicating that the rats were in constant dioestrum. Notably, the vaginal cells of OVX rats treated with EB or YPBYD were keratinized after about 3 and 2 days of treatment, respectively, which indicated that the treated OVX rats gradually entered the oestrus state. These results revealed that the YPBYD treatment had a certain therapeutic effect on sexual dysfunction in rats.

### Effect of YPBYD on the serum levels of cAMP and E2

**Figure 2.**
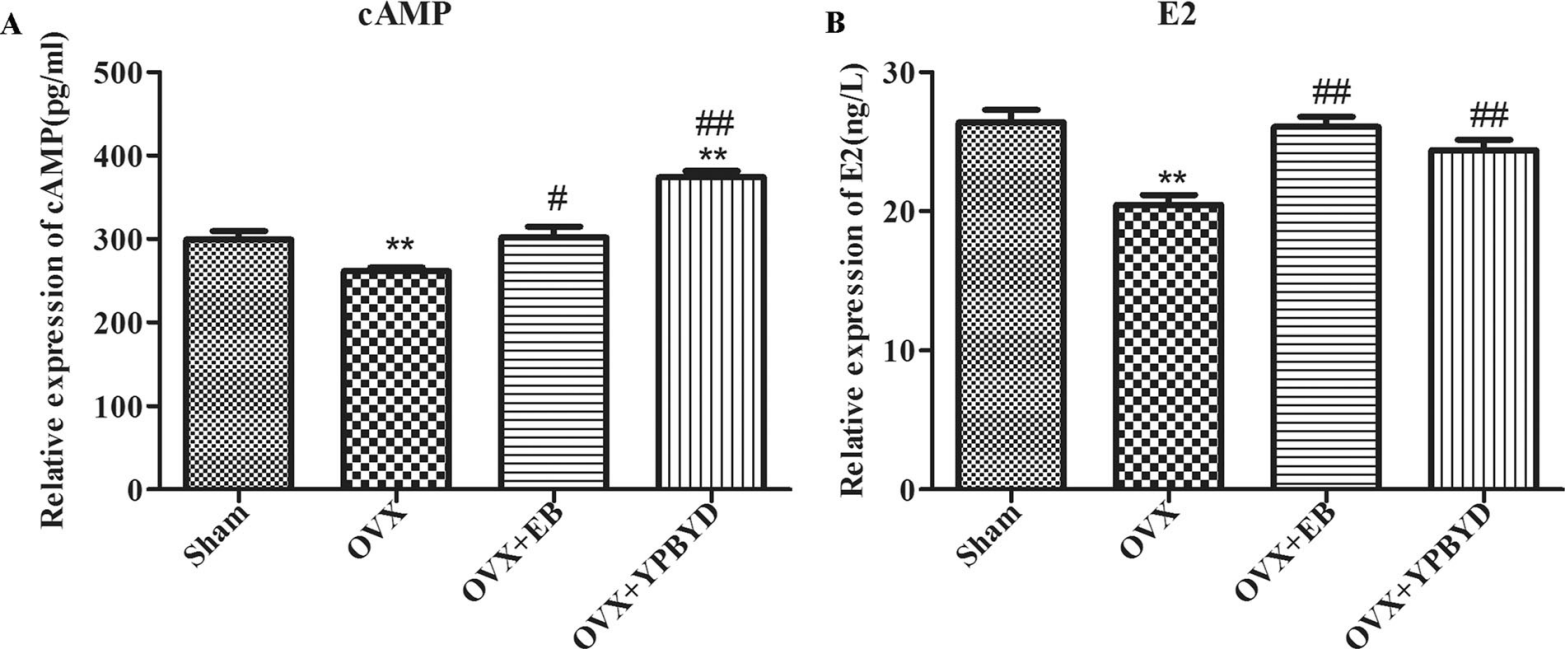
Effect of YPBYD treatment on the levels of cyclic adenosine monophosphate (cAMP) and oestradiol (E2). (A) Serum levels of cAMP from rats. (B) Serum levels of E2 from rats. Data analysis between two groups was performed using ANOVA and the Newman–Keuls multiple comparison test. **p < 0.01 compared with Sham group, ^#^*p* < 0.05 and ^##^*p* < 0.01 compared with OVX group.

Compared with the rats in the Sham group (299.39 ± 22.40 pg/mL), those in the OVX group had lower serum levels of cAMP (261.40 ± 9.21 pg/mL, *p* < 0.01). After treatment with EB or YPBYD, the level of cAMP in rats significantly increased compared with that in the OVX group (EB: 301.91 ± 25.68 pg/mL, *p* < 0.05; YPBYD: 373.75 ± 17.37 pg/mL, *p* < 0.01, Figure 2(A)). In addition, the level of E2 in the OVX group was significantly decreased compared with that in the Sham group (20.45 ± 1.61 ng/L vs. 26.38 ± 1.61 ng/L, *p* < 0.01). After the 4-week treatment, the serum level of E2 in OVX rats returned to normal (EB: 25.05 ± 1.79 ng/L, *p* < 0.01; YPBYD: 24.38 ± 1.71 ng/L, *p* < 0.01, Figure 2(B)).

### Overview of 16S rRNA sequencing data

To explore the effect of YPBYD on the gut microbiota of OVX rats, the stool samples were collected for 16S rRNA sequencing. A total of 959,204 sequences and 397,208,516 bases were obtained from the 24 stool samples. The average length of the sequences was 414 bp, and the results of each sample are presented in Table 1.

**Table 1.** 16S rRNA sequencing results of each sample.

Sample	Seq_num	Base_num	Mean_length	Min_length	Max_length
C1	42543	17825261	418.994	219	489
C2	36988	15367284	415.4667	317	432
C3	38225	16066226	420.3068	211	432
C4	42650	17694270	414.8715	283	506
C5	40353	16776944	415.7546	252	489
C6	36462	15104378	414.2498	363	441
C_1_1	36704	15280979	416.3301	233	432
C_1_2	40812	16844325	412.7297	277	433
C_1_3	37852	15574626	411.4611	293	433
C_1_4	39222	16163179	412.0947	254	480
C_1_5	49592	20283219	409.0018	252	447
C_1_6	37266	15446380	414.4899	291	431
C_1	36054	15052339	417.4943	265	440
C_2	39839	16627037	417.3558	317	477
C_3	38436	16001795	416.3231	270	433
C_4	34516	14304805	414.4398	292	434
C_5	43189	17736398	410.6693	233	434
C_6	39419	16053294	407.2476	317	434
T1	40411	16819390	416.2082	245	442
T2	39069	16035045	410.4289	232	443
T3	44067	18082153	410.3332	277	455
T4	46018	19078368	414.5849	254	443
T5	40387	16896128	418.3556	300	489
T6	39130	16094693	411.3134	259	489

C1-6: Sham group; C_1_1-6: OVX group; C_1-6: OVX + EB group; T1-6: OVX + YPBYD.

### Analysis of microbiota diversity and community composition

**Figure 3.**
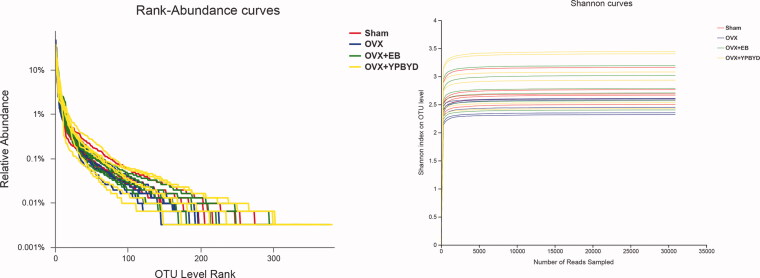
Rank abundance curve and rarefaction curve of the 16S rRNA sequencing data. Red represents the Sham group, blue represents the OVX group, green represents the OVX + EB group and yellow represents the OVX + YPBYD group.

**Figure 4.**
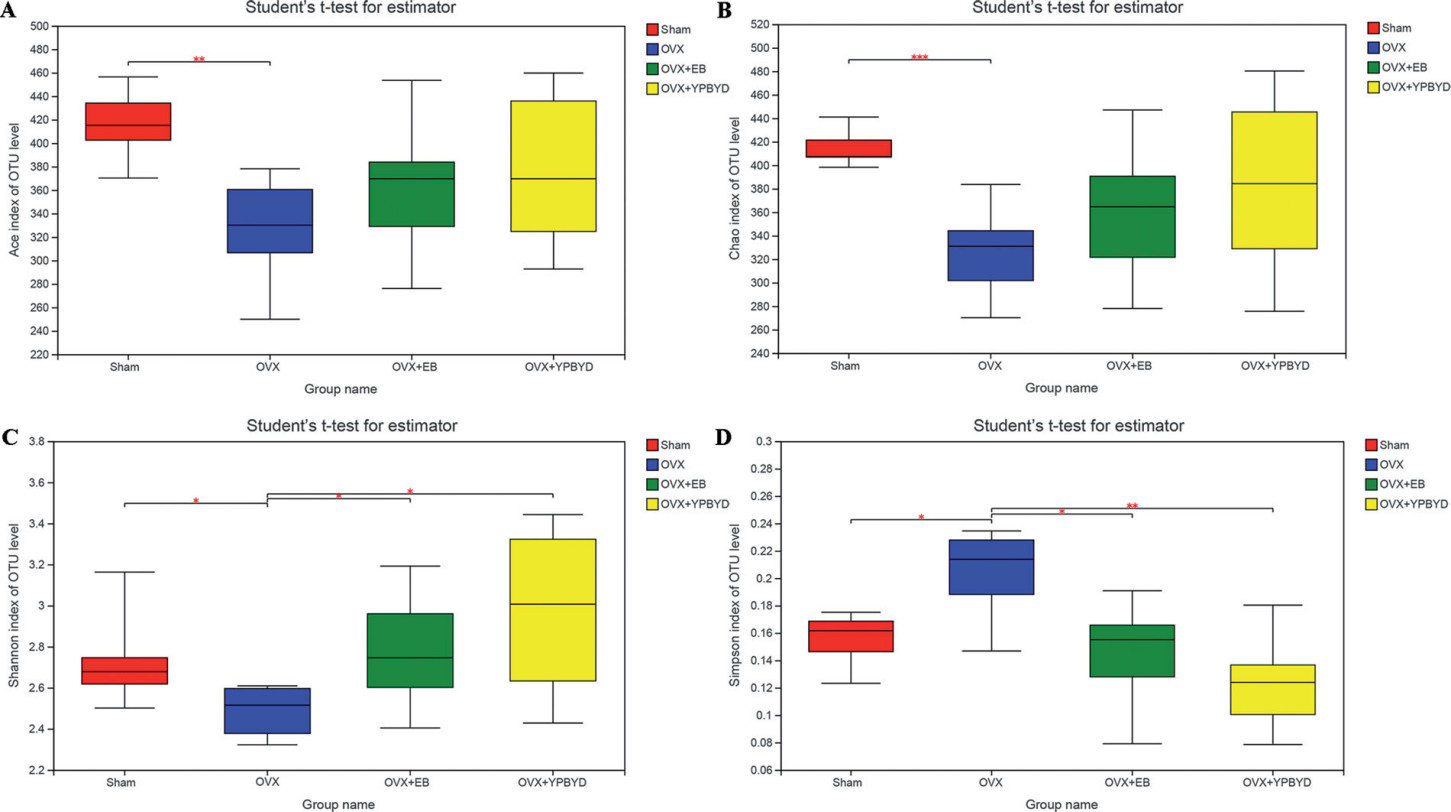
Alpha diversity analysis of gut microbial community structure in each group. (A) Ace index. (B) Chao index. (C) Shannon index. (D) Simpson index. Red represents the Sham group, blue represents the OVX group, green represents the OVX + EB group and yellow represents the OVX + YPBYD group. *p < 0.05, **p < 0.01 and ***p < 0.001.

**Figure 5.**
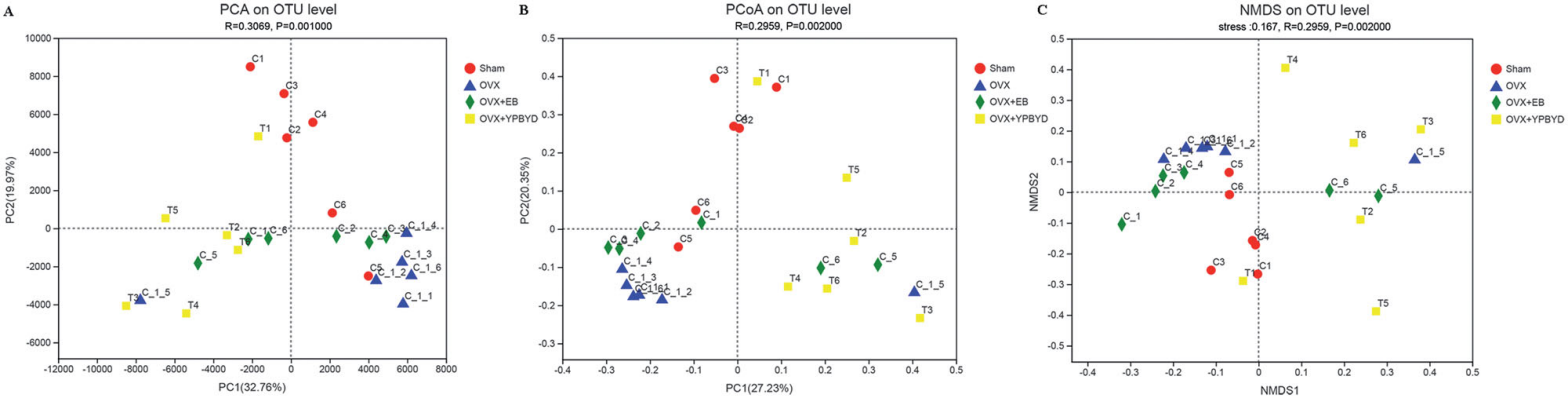
Beta diversity analysis of gut microbial community structure in each group. (A) PCA plot. (B) PCoA plot. (C) NMDS plot. Red represents the Sham group, blue represents the OVX group, green represents the OVX + EB group and yellow represents the OVX + YPBYD group.

The rank abundance curve and rarefaction curve showed a flattening trend, indicating that the species distribution was uniform and the sequencing data were large enough to reflect the majority of microbial diversity information in the samples (Figure 3). Next, the alpha and beta diversity analyses were conducted. Ace and Chao indexes were used to estimate the richness of species, while Simpson and Shannon indexes were used to assess the diversity of microbes. Compared with the Sham group, the Ace, Chao and Shannon indexes of the OVX group were significantly lower (*p* < 0.05, Figure 4(A–C)), while the Simpson index was significantly higher (*p* < 0.05, Figure 4(D)). These results revealed that the OVX group had lower richness and microbial diversity than the Sham group. Meanwhile, the diversity of microbiota slightly improved after EB or YPBYD treatment, indicating that YPBYD could relieve the dysregulation of intestinal flora. In addition, the beta diversity (PCA, PCoA and NMDS plots) showed that the sample in each group had obvious clustering and the bacterial structure of the Sham and OVX groups was obviously separated (Figure 5(A–C)).

**Figure 6.**
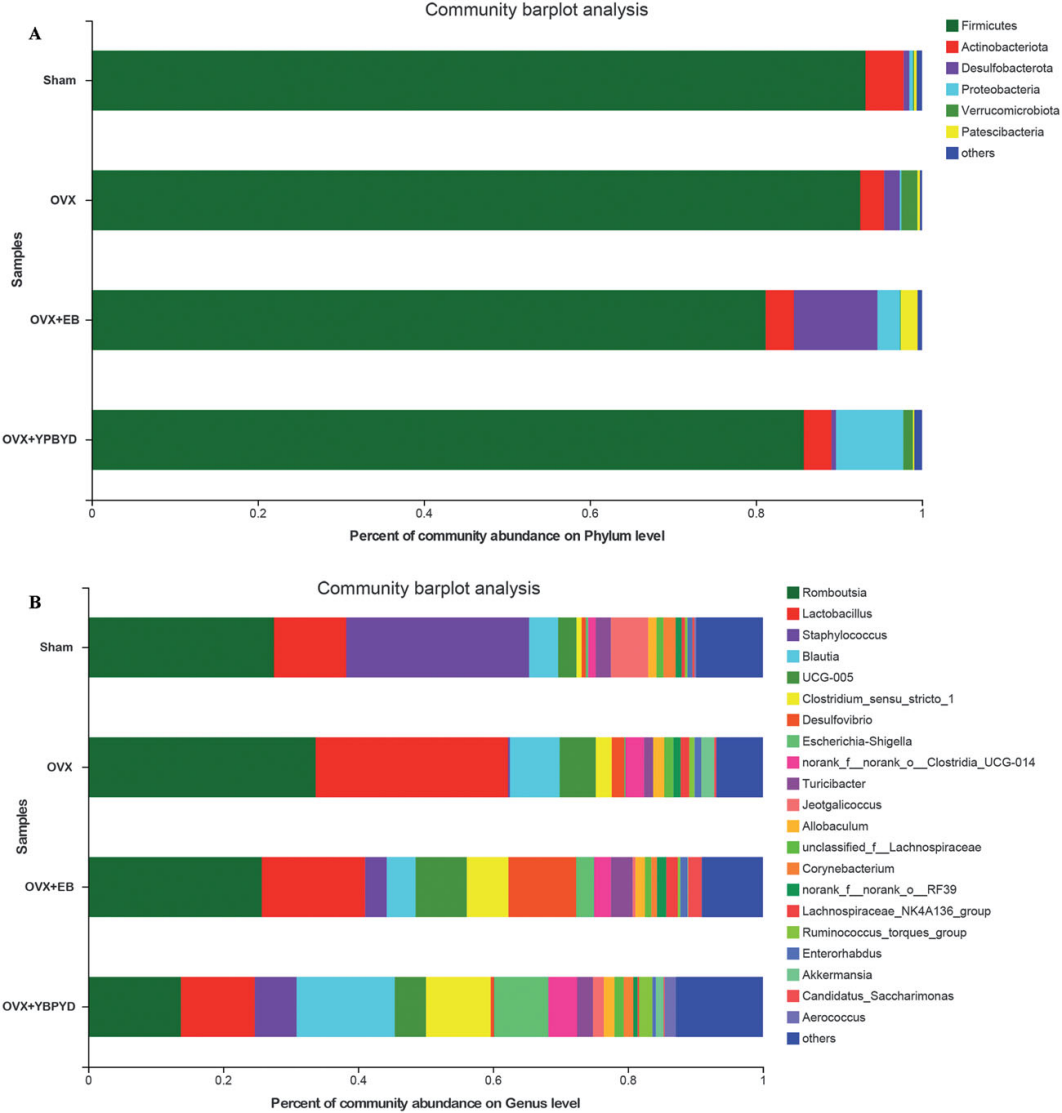
Effect of YPBYD treatment on the gut microbial composition of rats. (A) Bacterial composition of the gut microbiotas at the phylum level. (B) Bacterial composition of the gut microbiotas at the genus level. The different coloured columns represent different species, while the length of the columns represents the size of the species.

Furthermore, the gut microbial composition at phylum and genus levels was analysed to observe the alterations in bacteria caused by YPBYD treatment. In brief, at the phylum level (Figure 6(A)), Firmicutes, Actinobacteriota, Desulfobacterota and Proteobacteria were the three dominant phyla in these four groups. In brief, the abundance levels of Firmicutes in Sham, OVX, OVX + EB and OVX + YPBYD groups were 93.19%, 92.57%, 81.91% and 85.78%, respectively. The abundance level of Actinobacteriota in the OVX group (2.87%) was lower than that in the Sham group (4.60%), and the relative abundance of it increased to 3.33% after YPBYD treatment. Compared with the Sham group (0.69%), the abundance level of Desulfobacterota in the OVX group increased (1.88%); meanwhile, the abundance level of Desulfobacterota decreased in the OVX + YPBYD group (0.55%) compared with that in the OVX group. In addition, the abundance level of Proteobacteria decreased in the OVX group (0.20%) compared with that in the Sham group (0.44%); by contrast, the abundance level of Proteobacteria increased after YPBYD treatment (8.10%).

At the genus level, *Romboutsia*, *Lactobacillus* and *Staphylococcus* were predominant genera in these groups (Figure 6(B)). Results revealed that the abundance levels of both *Romboutsia* and *Lactobacillus* in the OVX group were greater than those in the Sham group. Following the YPBYD treatment, the abundance levels of *Romboutsia* and *Lactobacillus* were lower in the OVX + YPBYD group than in the OVX group. Meanwhile, the abundance level of *Staphylococcus* decreased in the OVX group compared with that in the Sham group; by contrast, the abundance level of *Staphylococcus* increased in the OVX + YPBYD group compared with that in the OVX group. These results suggested that YPBYD might play a therapeutic role by partially restoring the dysfunctional gut microbiota.

### Results of differential analysis of gut microbiota altered by YPBYD treatment

**Figure 7.**
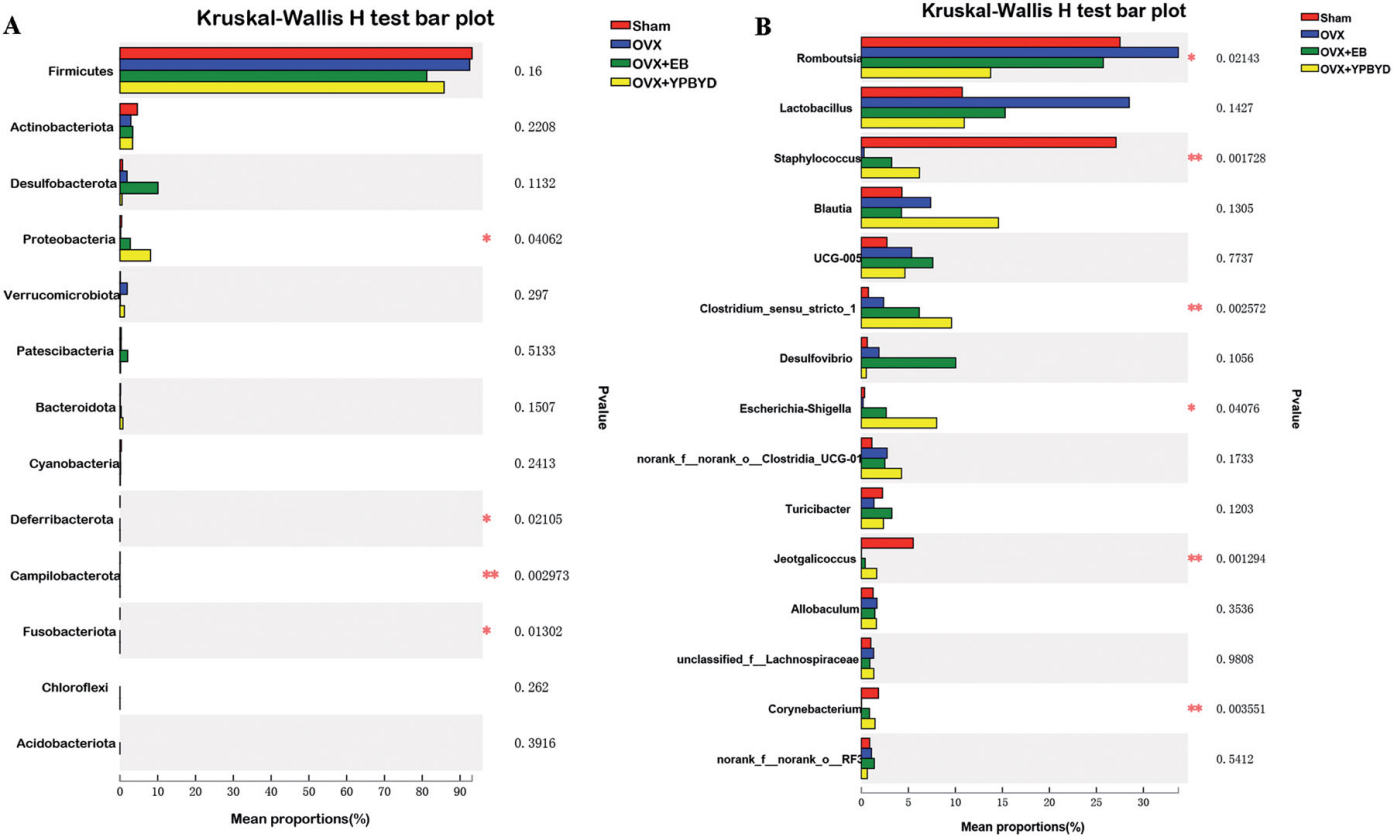
Differential analysis of gut microbiotas altered by YPBYD treatment. *p < 0.05, **p < 0.01.

To further screen the significant differences in the gut microbiotas altered by YPBYD treatment, differential analysis and LEfSe analysis were conducted. As shown in Figure 7, several significantly changed phyla, such as Proteobacteria, Deferribacterota and Campilobacterota (Figure 7(A)), as well as some significant altered genera, such as *Romboutsia*, *Staphylococcus*, *Jeotgalicoccus* and *Corynebacterium* (Figure 7(B)), were identified. We observed that the abundance levels of Proteobacteria as well as those of *Staphylococcus*, *Jeotgalicoccus* and *Corynebacterium* was decreased in the OVX group compared with that in the Sham group, while the abundance levels of these microbes increased in the treatment groups (OVX + EB and OVX + YPBYD). In addition, the abundance level of *Romboutsia* was significantly increased in the OVX group compared with that in the Sham group, and the abundance level of *Romboutsia* obviously decreased in the OVX + YPBYD group compared with that in the OVX group.

**Figure 8.**
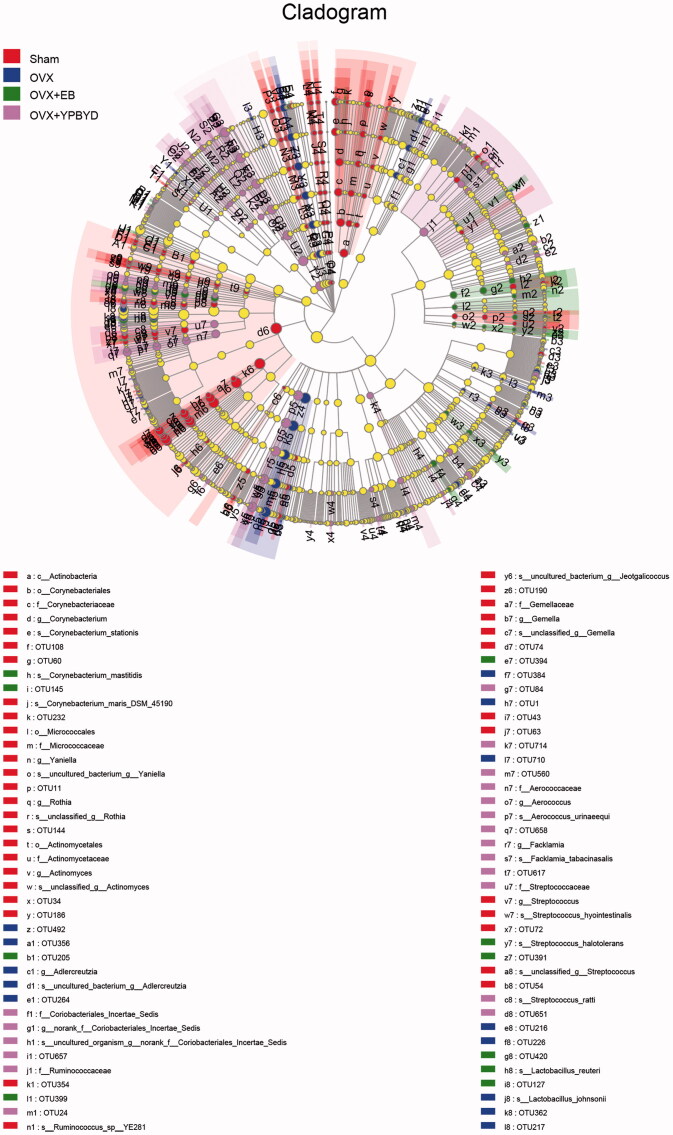
Linear discriminant analysis effect size cladograms for the comparisons of the four study groups. Red represents the Sham group, blue represents the OVX group, green represents the OVX + EB group and pink represents the OVX + YPBYD group.

Furthermore, 359 taxa among these four groups were identified using LEfSe analysis (Figure 8), and the LDA scores ranged from 0.0 to 5.5. Results indicated that the dominant bacteria in the Sham group were *f_Staphylococcaceae* and *g_Staphylococcus*; those in the OVX group were *f_Peptostreptococcaceae* and *o_Peptostreptococcales_Tissierellales*; those in the OVX + EB group were dominated by *s_Lactobacillus_reuteri* and *g_Enterococcus*; and those in the OVX + YPBYD group were *f_Clostridiaceae* and *o_Clostridiales*.

### Results of predicted microbial function

**Figure 9.**
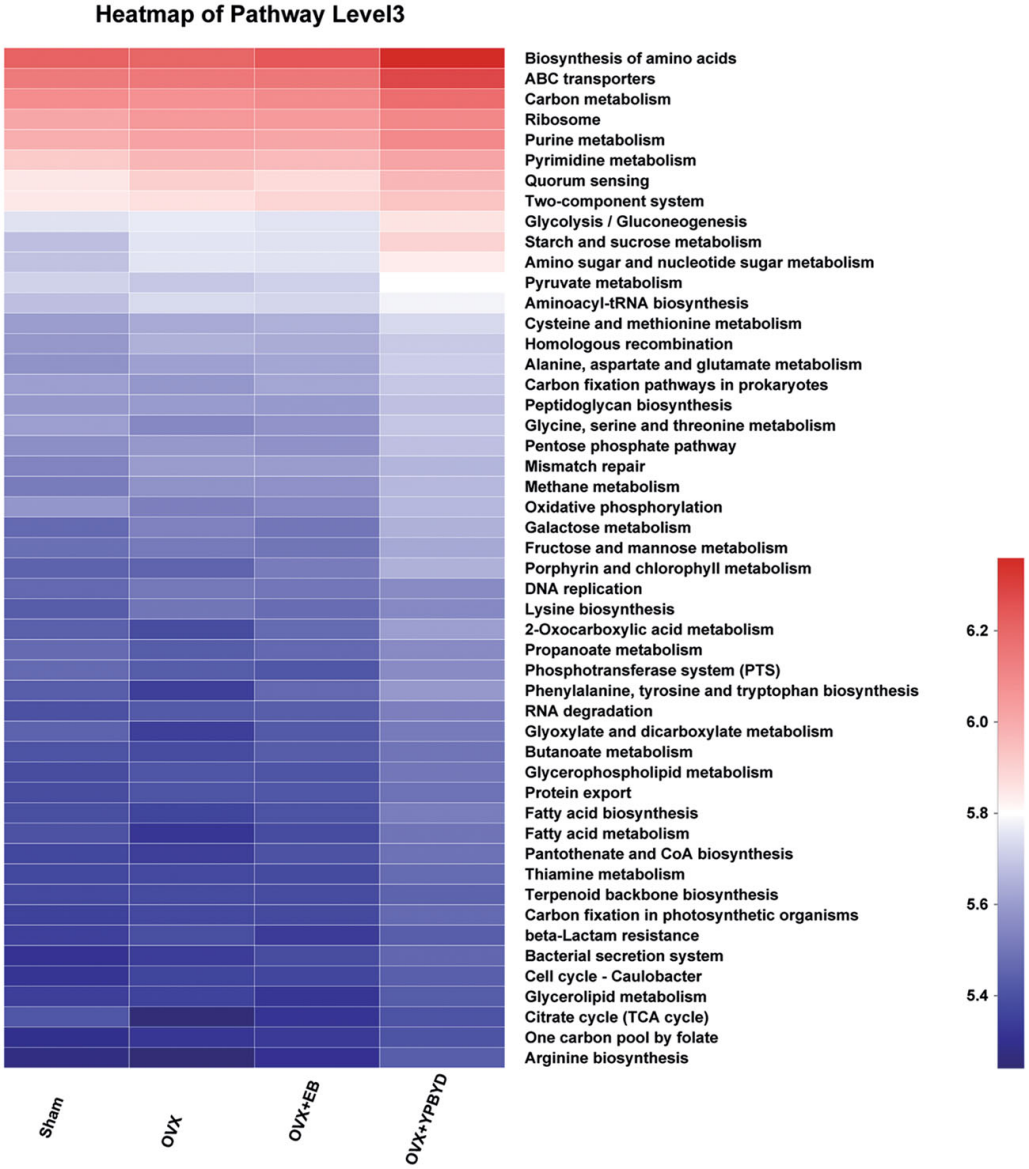
Microbial community function was predicted by PICRUst.

We determined the KEGG pathway involved in the microbiota function using PICRUst. A total of 50 KEGG pathways, which were divided into six categories (metabolism, generic information processing, environmental information processing, cellular processes, human diseases and organismal systems) were identified. As shown in Figure 6, the microbes related to YPBYD treatment were mainly enriched in the biosynthesis of amino acids, pyruvate metabolism, phenylalanine, tyrosine and tryptophan biosynthesis, fatty acid metabolism and citrate cycle (Figure 9).

## Discussion

Sexual function depends on the complex interaction of physical, psychosocial and neurobiological factors. It may be affected centrally (brain) or peripherally (genitals) or both, and may affect any or all stages of the sexual response cycle (Clayton and Valladares Juarez 2017). The balance between excitatory and inhibitory neuromodulation process is thought to produce specific sexual responses. It is believed that sexual dysfunction is due to overactive inhibition, overactive excitement or a combination of both (Faubion and Rullo 2015). Accumulated evidence indicates that gut microbiota can modulate neurotransmitter and plays roles in the development and impairment of the central nervous system (Strandwitz 2018). Thus, it is necessary to explore the role of intestinal microbiotas in the pathogenesis of sexual dysfunction and to screen the microbe with potential therapeutic effects in this disease. In the present study, we explored the changes in gut microbiota of female rat model with sexual dysfunction. Results showed that the serum levels of cAMP and E2 in OVX rats were significantly decreased compared with those in Sham rats; meanwhile, the levels of these indexes were markedly increased after EB and YPBYD treatment. The relative abundance of microbes such as *Proteobacteria*, *Romboutsia* and *Staphylococcus* was significantly changed in the OVX rats during the period of YPBYD treatment.

Results of the vaginal smear showed that the OVX rats did not have a complete oestrus cycle, and most of the vaginal epithelial cells were leukocytes, indicating that the rats were in the dioestrus state. These findings indicated that the model of sexual dysfunction was successfully constructed. Moreover, ELISA result revealed that the level of E2 in the OVX group was lower than that in the Sham group. E2 is implicated as one of the steroids important for modulating the sexual desire of women and is crucial to the sexual motivation and arousal of women (Rao et al. 2015). Previous study found that oestrogen therapy could increase the postmenopausal women’s sexual desire by producing periovulatory levels of circulating E2 (Cappelletti and Wallen 2016). In addition, the use of hormonal contraceptives reduced the neural response of women to sexual stimulation by decreasing the level of several hormones such as E2, which could possibly lead to sexual dysfunction (Casado-Espada et al. 2019). These studies highlighted the important regulatory role of E2 in sexual response. In this experiment, the level of E2 in OVX rats was increased after YPBYD treatment; staining results showed that the YPBYD-treated OVX rats gradually entered the oestrus state, which further confirmed that YPBYD treatment improved the symptoms of sexual dysfunction.

Sexual dysfunction can adversely affect the QoL, self-esteem and interpersonal relationships, and may lead to psychopathological disorders (Jaafarpour et al. 2013). Meanwhile, eating disorders are closely related to the occurrence of sexual dysfunction (Castellini et al. 2019). To our knowledge, the underlying relationship between microbes and psychopathological state as well as dietary habit has been recognized (Rinninella et al. 2019). Thus, we explored the direct association between gut microbiota and sexual dysfunction (Bersani et al. 2020). Our results showed that the species evenness and richness was significantly decreased in the OVX group than in the Sham group; meanwhile, YPBYD treatment markedly enhanced the richness and diversity of gut microbiota in OVX rats, indicating that YPBYD had the ability to restore the structure of the dysfunctional intestinal microbiota. Notably, we observed that the abundance levels of Proteobacteria and *Staphylococcus* in the OVX group were lower than those in the Sham group; however, YPBYD treatment could restore these changes. Previous study indicated that the abundance level of Proteobacteria decreased in the OVX mice, which was associated with their susceptibility to metabolic syndrome (MS) and metabolic endotoxaemia (Kaliannan et al. 2018). Di Francesco et al. (2019) reported that MS was closely linked to female sexual dysfunctions, such as sexual desire disorder, orgasm disorder and decreased satisfaction. A previous study on *Staphylococcus* indicated that *Staphylococcus aureus* induced an increase in the levels of prostaglandin E(2) [PGE92)] in the hypothalamus (Martins et al. 2012), and the hypothalamus and limbic systems were involved in sexual arousal (Harsh and Clayton 2018). Moreover, PGE(2) might be used as a novel strategy for the treatment of erectile dysfunction (El Melegy et al. 2005), and PGE(2) could serve a crucial role in improving sexual pain caused by endometriosis (Peng et al. 2018). These studies emphasized that these microbiotas were indirectly linked to the occurrence of sexual dysfunction, and YPBYD treatment might provide therapeutic effects by altering the abundance of these microbes. Furthermore, the relative abundance of *Romboutsia* was significantly changed after YPBYD treatment, which also attracted our attention. Zeng et al. (2019) demonstrated that several microbes such as *Romboutsia* were positively correlated with body weight and serum lipids, which might be considered as gut microbiota markers for obesity-related metabolic abnormalities. Obesity could increase the risk of erectile dysfunction in men and the prevalence of sexual dysfunction in women (Esposito et al. 2008). Taken together, *Romboutsia* might play a role in the pathogenesis of sexual dysfunction.

We also predicted the function of identified microbiotas, and results showed that microbes were significantly involved in tyrosine and tryptophan biosynthesis as well as fatty acid metabolism. Tyrosine kinases had beneficial effects on erection and sexual function (Holland et al. 2017). Meanwhile, tryptophan had the ability to improve the sexual quality in patients with premature ejaculation (Sansalone et al. 2016). In addition, disturbed fatty acid metabolism might be regarded as a potential marker of vasculogenic erectile dysfunction (Ben Khedher et al. 2017). Thus, we speculated that these functions might also serve important roles in the occurrence of female sexual dysfunction.

This study is the first to explore the mechanism of YPBYD treatment, suggesting that YPBYD can improve the symptoms of sexual dysfunction, possibly by modulating the gut microbiota in OVX rats. However, there are some limitations in this study. First, our research has incomplete data. The study is only conducted in female rats with sexual dysfunction, and male rats are not included. Second, the specific association between these identified microbiotas and sexual dysfunction-related clinical characteristics has not been investigated. Finally, the gut microbiota of rats is different from those of human, so whether these screened microbes play a role in human sexual dysfunction needs to be further investigated. Therefore, a follow-up study is warranted to assess whether the obtained microbes could be used as markers and therapeutic targets of sexual dysfunction.

## Conclusions

This study is the first to provide evidence confirming that intestinal microbiotas play a key role in the pathogenesis and YPBYD treatment of sexual dysfunction. Results showed that sexual dysfunction caused by ovariectomy could lead to the changes in the diversity and structure of intestinal microbiota in rats, and YPBYD treatment could partially reverse these alterations. OVX rats had a higher abundance of *Romboutsia* and lower abundance of Proteobacteria and *Staphylococcus* compared with Sham group; meanwhile, YPBYD treatment could decrease the level of *Romboutsia* and increase the abundance of Proteobacteria and *Staphylococcus*. Moreover, YPBYD influenced the KEGG pathways such as tyrosine and tryptophan biosynthesis as well as fatty acid metabolism. Together, these findings indicated that the effects of YPBYD on female sexual dysfunction might depend on its regulation of gut microbiota.

## Author contributions

Conception and research design: Yangyun Wang and Guowei Shi; acquisition of data: Chaoliang Shi, Wandong Yu and Wei Jiao; analysis and interpretation of data: Chaoliang Shi, Wandong Yu and Wei Jiao; statistical analysis: Chaoliang Shi, Wandong Yu and Wei Jiao; obtaining funding: Guowei Shi; drafting the manuscript: Yangyun Wang; revision of the manuscript for important intellectual content: Guowei Shi. All authors read and approved the final manuscript.

## Data Availability

All data generated or analysed during this study are included in this published article.
